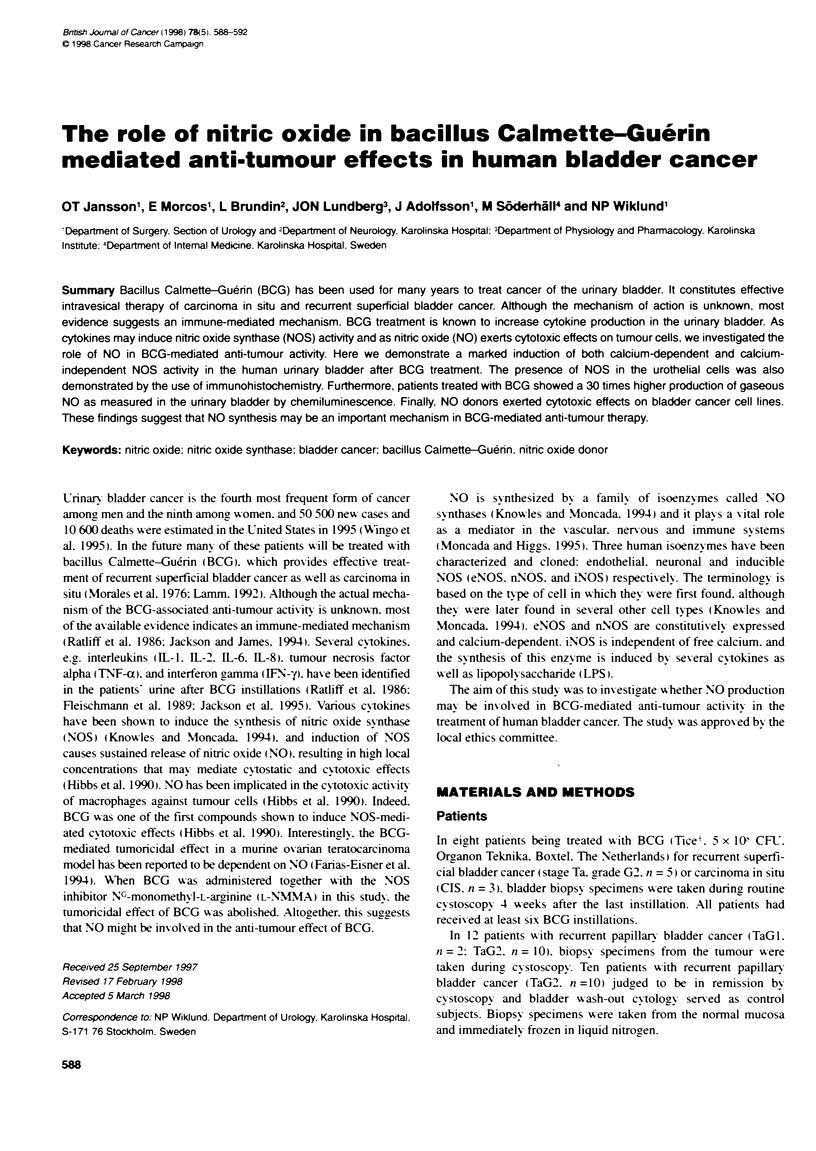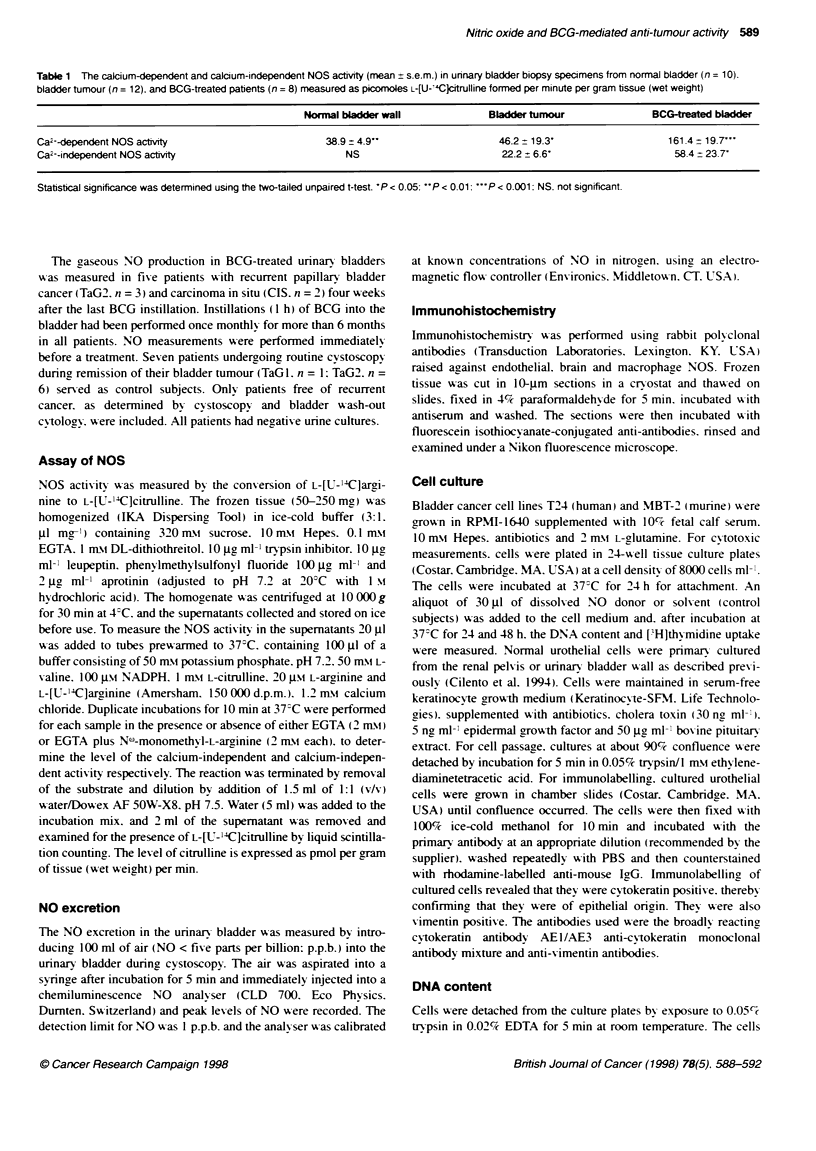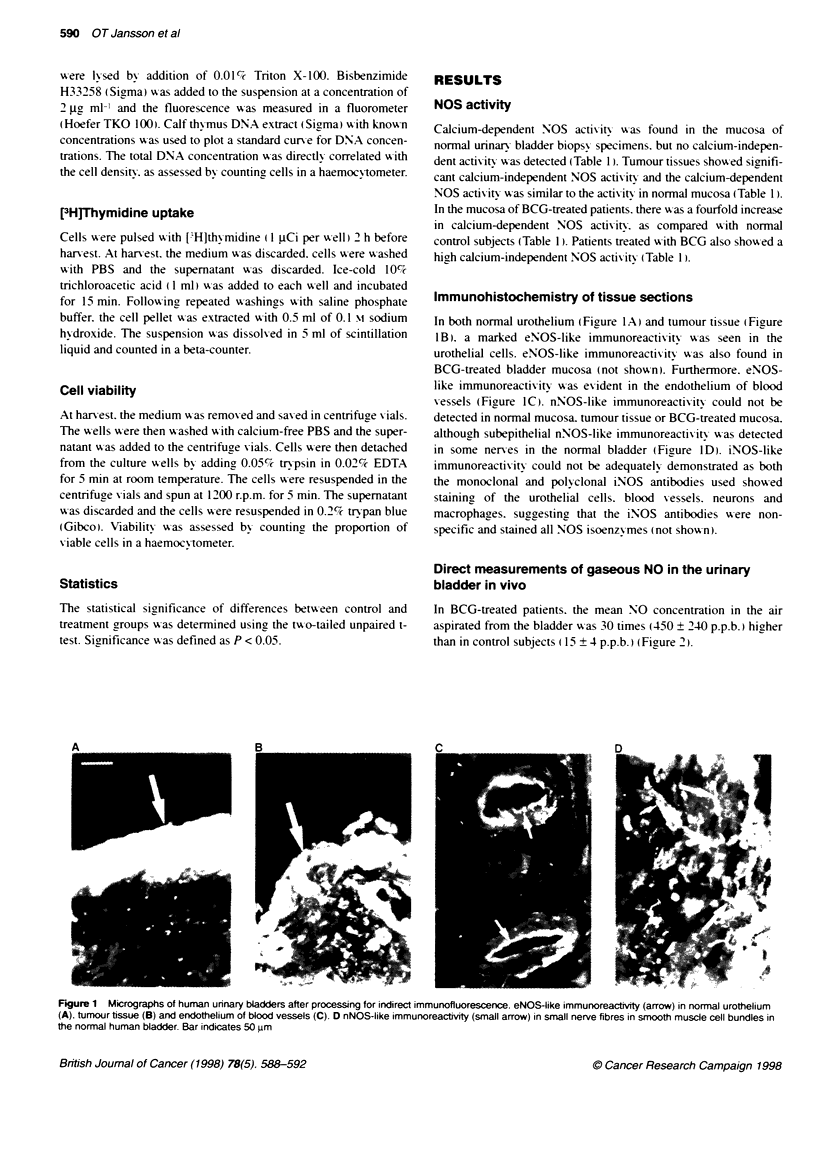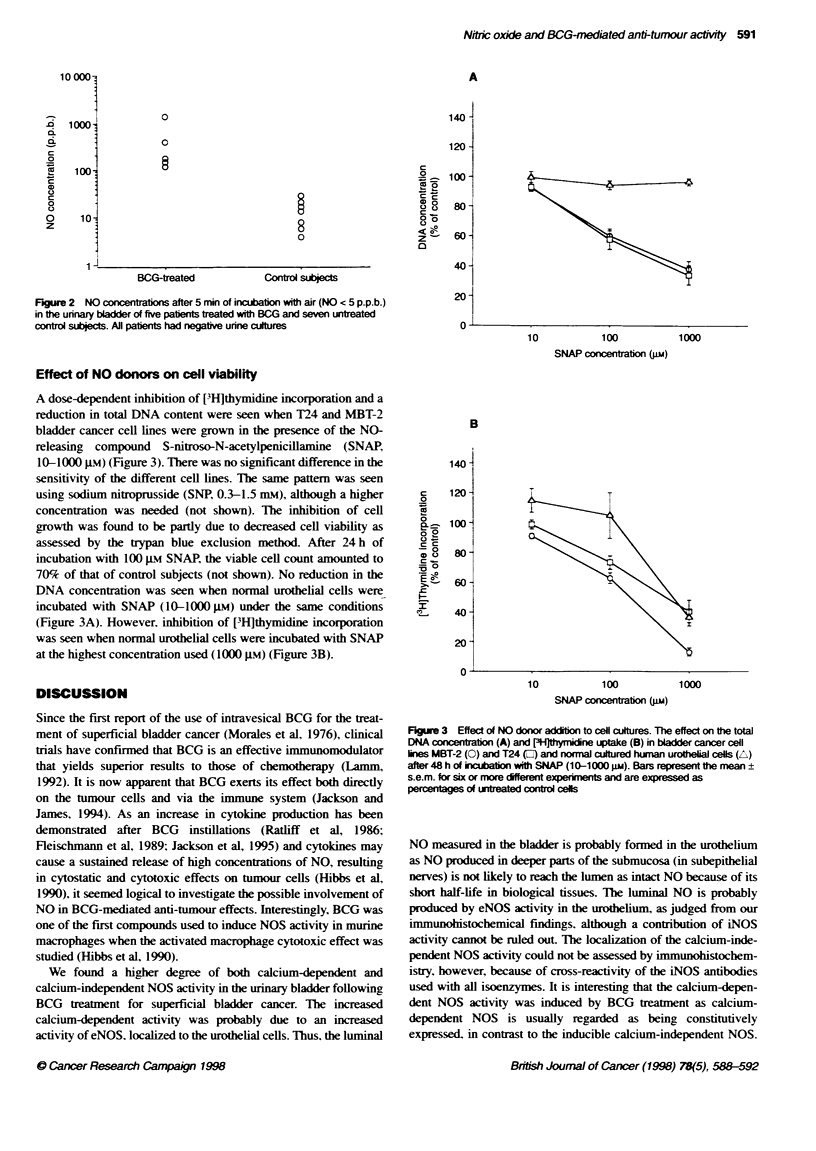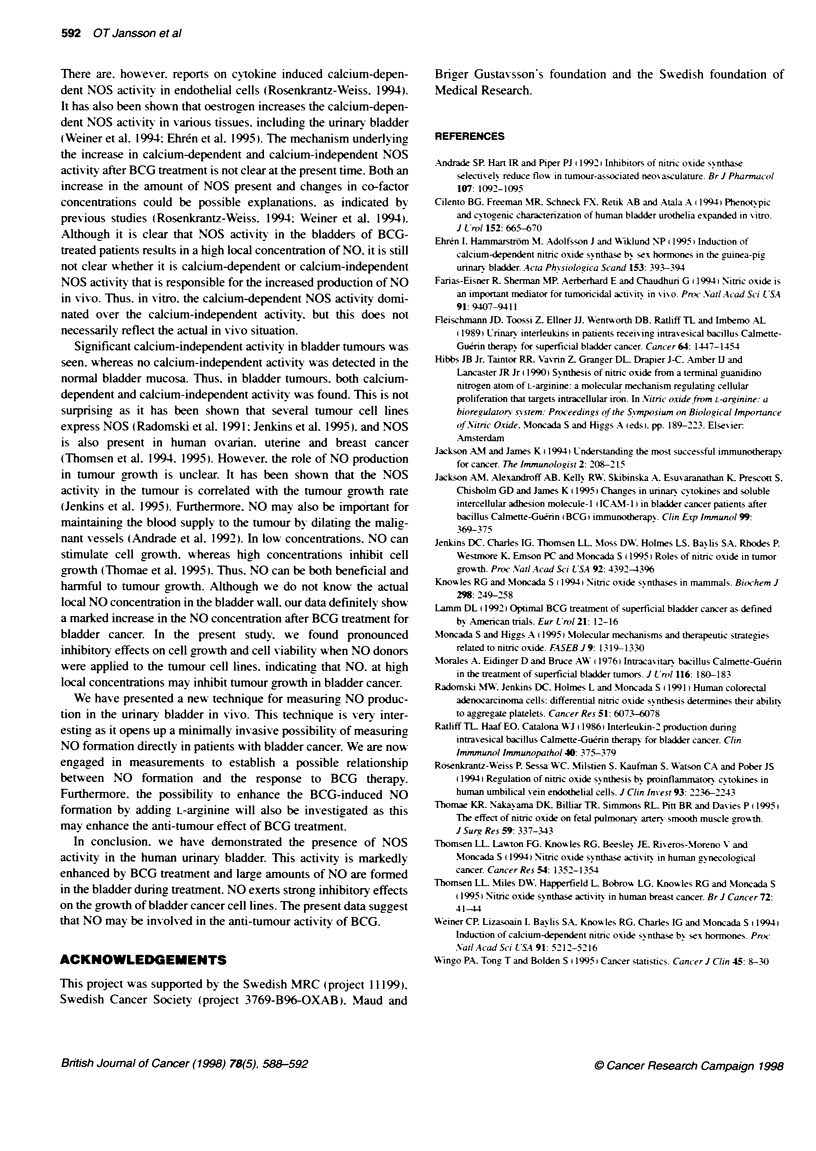# The role of nitric oxide in bacillus Calmette-Guérin mediated anti-tumour effects in human bladder cancer.

**DOI:** 10.1038/bjc.1998.545

**Published:** 1998-09

**Authors:** O. T. Jansson, E. Morcos, L. Brundin, J. O. Lundberg, J. Adolfsson, M. Söderhäll, N. P. Wiklund

**Affiliations:** Department of Surgery, Karolinska Hospital, Sweden.

## Abstract

**Images:**


British Journal of Cancer (1998) 78(5), 588–592
© 1998 Cancer Research Campaign

### The role of nitric oxide in bacillus Calmette–Guérin mediated anti-tumour effects in human bladder cancer

OT Jansson[1], E Morcos[1], L Brundin[2], JON Lundberg[3], J Adolfsson[1], M Söderhäll[4] and NP Wiklund[1]

[1]Department of Surgery, Section of Urology and [2]Department of Neurology, Karolinska Hospital; [3]Department of Physiology and Pharmacology, Karolinska Institute; [4]Department of Internal Medicine, Karolinska Hospital, Sweden

**Summary** Bacillus Calmette–Guérin (BCG) has been used for many years to treat cancer of the urinary bladder. It constitutes effective intravesical therapy of carcinoma in situ and recurrent superficial bladder cancer. Although the mechanism of action is unknown, most evidence suggests an immune-mediated mechanism. BCG treatment is known to increase cytokine production in the urinary bladder. As cytokines may induce nitric oxide synthase (NOS) activity and as nitric oxide (NO) exerts cytotoxic effects on tumour cells, we investigated the role of NO in BCG-mediated anti-tumour activity. Here we demonstrate a marked induction of both calcium-dependent and calcium-independent NOS activity in the human urinary bladder after BCG treatment. The presence of NOS in the urothelial cells was also demonstrated by the use of immunohistochemistry. Furthermore, patients treated with BCG showed a 30 times higher production of gaseous NO as measured in the urinary bladder by chemiluminescence. Finally, NO donors exerted cytotoxic effects on bladder cancer cell lines. These findings suggest that NO synthesis may be an important mechanism in BCG-mediated anti-tumour therapy.

**Keywords:** nitric oxide; nitric oxide synthase; bladder cancer; bacillus Calmette–Guérin, nitric oxide donor

Urinary bladder cancer is the fourth most frequent form of cancer among men and the ninth among women, and 50 500 new cases and 10 600 deaths were estimated in the United States in 1995 (Wingo et al, 1995). In the future many of these patients will be treated with bacillus Calmette–Guérin (BCG), which provides effective treatment of recurrent superficial bladder cancer as well as carcinoma in situ (Morales et al, 1976; Lamm, 1992). Although the actual mechanism of the BCG-associated anti-tumour activity is unknown, most of the available evidence indicates an immune-mediated mechanism (Ratliff et al, 1986; Jackson and James, 1994). Several cytokines, e.g. interleukins (IL-1, IL-2, IL-6, IL-8), tumour necrosis factor alpha (TNF-α), and interferon gamma (IFN-γ), have been identified in the patients' urine after BCG instillations (Ratliff et al, 1986; Fleischmann et al, 1989; Jackson et al, 1995). Various cytokines have been shown to induce the synthesis of nitric oxide synthase (NOS) (Knowles and Moncada, 1994), and induction of NOS causes sustained release of nitric oxide (NO), resulting in high local concentrations that may mediate cytostatic and cytotoxic effects (Hibbs et al, 1990). NO has been implicated in the cytotoxic activity of macrophages against tumour cells (Hibbs et al, 1990). Indeed, BCG was one of the first compounds shown to induce NOS-mediated cytotoxic effects (Hibbs et al, 1990). Interestingly, the BCG-mediated tumoricidal effect in a murine ovarian teratocarcinoma model has been reported to be dependent on NO (Farias-Eisner et al, 1994). When BCG was administered together with the NOS inhibitor $N^G$-monomethyl-L-arginine (L-NMMA) in this study, the tumoricidal effect of BCG was abolished. Altogether, this suggests that NO might be involved in the anti-tumour effect of BCG.

*Received 25 September 1997*
*Revised 17 February 1998*
*Accepted 5 March 1998*

*Correspondence to:* NP Wiklund, Department of Urology, Karolinska Hospital, S-171 76 Stockholm, Sweden

NO is synthesized by a family of isoenzymes called NO synthases (Knowles and Moncada, 1994) and it plays a vital role as a mediator in the vascular, nervous and immune systems (Moncada and Higgs, 1995). Three human isoenzymes have been characterized and cloned: endothelial, neuronal and inducible NOS (eNOS, nNOS, and iNOS) respectively. The terminology is based on the type of cell in which they were first found, although they were later found in several other cell types (Knowles and Moncada, 1994). eNOS and nNOS are constitutively expressed and calcium-dependent. iNOS is independent of free calcium, and the synthesis of this enzyme is induced by several cytokines as well as lipopolysaccharide (LPS).

The aim of this study was to investigate whether NO production may be involved in BCG-mediated anti-tumour activity in the treatment of human bladder cancer. The study was approved by the local ethics committee.

### MATERIALS AND METHODS

### Patients

In eight patients being treated with BCG (Tice®, 5 × 10⁸ CFU, Organon Teknika, Boxtel, The Netherlands) for recurrent superficial bladder cancer (stage Ta, grade G2, $n = 5$) or carcinoma in situ (CIS, $n = 3$), bladder biopsy specimens were taken during routine cystoscopy 4 weeks after the last instillation. All patients had received at least six BCG instillations.

In 12 patients with recurrent papillary bladder cancer (TaG1, $n = 2$; TaG2, $n = 10$), biopsy specimens from the tumour were taken during cystoscopy. Ten patients with recurrent papillary bladder cancer (TaG2, $n = 10$) judged to be in remission by cystoscopy and bladder wash-out cytology served as control subjects. Biopsy specimens were taken from the normal mucosa and immediately frozen in liquid nitrogen.

**Table 1** The calcium-dependent and calcium-independent NOS activity (mean ± s.e.m.) in urinary bladder biopsy specimens from normal bladder ($n$ = 10), bladder tumour ($n$ = 12), and BCG-treated patients ($n$ = 8) measured as picomoles L-[U-$^{14}$C]citrulline formed per minute per gram tissue (wet weight)

| | Normal bladder wall | Bladder tumour | BCG-treated bladder |
|---|---|---|---|
| $Ca^{2+}$-dependent NOS activity | 38.9 ± 4.9** | 46.2 ± 19.3* | 161.4 ± 19.7*** |
| $Ca^{2+}$-independent NOS activity | NS | 22.2 ± 6.6* | 58.4 ± 23.7* |

Statistical significance was determined using the two-tailed unpaired t-test. *$P < 0.05$; **$P < 0.01$; ***$P < 0.001$; NS, not significant.

The gaseous NO production in BCG-treated urinary bladders was measured in five patients with recurrent papillary bladder cancer (TaG2, $n$ = 3) and carcinoma in situ (CIS, $n$ = 2) four weeks after the last BCG instillation. Instillations (1 h) of BCG into the bladder had been performed once monthly for more than 6 months in all patients. NO measurements were performed immediately before a treatment. Seven patients undergoing routine cystoscopy during remission of their bladder tumour (TaG1, $n$ = 1; TaG2, $n$ = 6) served as control subjects. Only patients free of recurrent cancer, as determined by cystoscopy and bladder wash-out cytology, were included. All patients had negative urine cultures.

### Assay of NOS

NOS activity was measured by the conversion of L-[U-$^{14}$C]arginine to L-[U-$^{14}$C]citrulline. The frozen tissue (50–250 mg) was homogenized (IKA Dispersing Tool) in ice-cold buffer (3:1, µl mg$^{-1}$) containing 320 mM sucrose, 10 mM Hepes, 0.1 mM EGTA, 1 mM DL-dithiothreitol, 10 µg ml$^{-1}$ trypsin inhibitor, 10 µg ml$^{-1}$ leupeptin, phenylmethylsulfonyl fluoride 100 µg ml$^{-1}$ and 2 µg ml$^{-1}$ aprotinin (adjusted to pH 7.2 at 20°C with 1 M hydrochloric acid). The homogenate was centrifuged at 10 000 $g$ for 30 min at 4°C, and the supernatants collected and stored on ice before use. To measure the NOS activity in the supernatants 20 µl was added to tubes prewarmed to 37°C, containing 100 µl of a buffer consisting of 50 mM potassium phosphate, pH 7.2, 50 mM L-valine, 100 µM NADPH, 1 mM L-citrulline, 20 µM L-arginine and L-[U-$^{14}$C]arginine (Amersham, 150 000 d.p.m.), 1.2 mM calcium chloride. Duplicate incubations for 10 min at 37°C were performed for each sample in the presence or absence of either EGTA (2 mM) or EGTA plus N$^{\omega}$-monomethyl-L-arginine (2 mM each), to determine the level of the calcium-independent and calcium-independent activity respectively. The reaction was terminated by removal of the substrate and dilution by addition of 1.5 ml of 1:1 (v/v) water/Dowex AF 50W-X8, pH 7.5. Water (5 ml) was added to the incubation mix, and 2 ml of the supernatant was removed and examined for the presence of L-[U-$^{14}$C]citrulline by liquid scintillation counting. The level of citrulline is expressed as pmol per gram of tissue (wet weight) per min.

### NO excretion

The NO excretion in the urinary bladder was measured by introducing 100 ml of air (NO < five parts per billion; p.p.b.) into the urinary bladder during cystoscopy. The air was aspirated into a syringe after incubation for 5 min and immediately injected into a chemiluminescence NO analyser (CLD 700, Eco Physics, Durnten, Switzerland) and peak levels of NO were recorded. The detection limit for NO was 1 p.p.b. and the analyser was calibrated at known concentrations of NO in nitrogen, using an electromagnetic flow controller (Environics, Middletown, CT, USA).

### Immunohistochemistry

Immunohistochemistry was performed using rabbit polyclonal antibodies (Transduction Laboratories, Lexington, KY, USA) raised against endothelial, brain and macrophage NOS. Frozen tissue was cut in 10-µm sections in a cryostat and thawed on slides, fixed in 4% paraformaldehyde for 5 min, incubated with antiserum and washed. The sections were then incubated with fluorescein isothiocyanate-conjugated anti-antibodies, rinsed and examined under a Nikon fluorescence microscope.

### Cell culture

Bladder cancer cell lines T24 (human) and MBT-2 (murine) were grown in RPMI-1640 supplemented with 10% fetal calf serum, 10 mM Hepes, antibiotics and 2 mM L-glutamine. For cytotoxic measurements, cells were plated in 24-well tissue culture plates (Costar, Cambridge, MA, USA) at a cell density of 8000 cells ml$^{-1}$. The cells were incubated at 37°C for 24 h for attachment. An aliquot of 30 µl of dissolved NO donor or solvent (control subjects) was added to the cell medium and, after incubation at 37°C for 24 and 48 h, the DNA content and [$^{3}$H]thymidine uptake were measured. Normal urothelial cells were primary cultured from the renal pelvis or urinary bladder wall as described previously (Cilento et al, 1994). Cells were maintained in serum-free keratinocyte growth medium (Keratinocyte-SFM, Life Technologies), supplemented with antibiotics, cholera toxin (30 ng ml$^{-1}$), 5 ng ml$^{-1}$ epidermal growth factor and 50 µg ml$^{-1}$ bovine pituitary extract. For cell passage, cultures at about 90% confluence were detached by incubation for 5 min in 0.05% trypsin/1 mM ethylenediaminetetracetic acid. For immunolabelling, cultured urothelial cells were grown in chamber slides (Costar, Cambridge, MA, USA) until confluence occurred. The cells were then fixed with 100% ice-cold methanol for 10 min and incubated with the primary antibody at an appropriate dilution (recommended by the supplier), washed repeatedly with PBS and then counterstained with rhodamine-labelled anti-mouse IgG. Immunolabelling of cultured cells revealed that they were cytokeratin positive, thereby confirming that they were of epithelial origin. They were also vimentin positive. The antibodies used were the broadly reacting cytokeratin antibody AE1/AE3 anti-cytokeratin monoclonal antibody mixture and anti-vimentin antibodies.

### DNA content

Cells were detached from the culture plates by exposure to 0.05% trypsin in 0.02% EDTA for 5 min at room temperature. The cells

© Cancer Research Campaign 1998

were lysed by addition of 0.01% Triton X-100. Bisbenzimide H33258 (Sigma) was added to the suspension at a concentration of 2 µg $ml^{-1}$ and the fluorescence was measured in a fluorometer (Hoefer TKO 100). Calf thymus DNA extract (Sigma) with known concentrations was used to plot a standard curve for DNA concentrations. The total DNA concentration was directly correlated with the cell density, as assessed by counting cells in a haemocytometer.

### [$^3$H]Thymidine uptake

Cells were pulsed with [$^3$H]thymidine (1 µCi per well) 2 h before harvest. At harvest, the medium was discarded, cells were washed with PBS and the supernatant was discarded. Ice-cold 10% trichloroacetic acid (1 ml) was added to each well and incubated for 15 min. Following repeated washings with saline phosphate buffer, the cell pellet was extracted with 0.5 ml of 0.1 M sodium hydroxide. The suspension was dissolved in 5 ml of scintillation liquid and counted in a beta-counter.

### Cell viability

At harvest, the medium was removed and saved in centrifuge vials. The wells were then washed with calcium-free PBS and the supernatant was added to the centrifuge vials. Cells were then detached from the culture wells by adding 0.05% trypsin in 0.02% EDTA for 5 min at room temperature. The cells were resuspended in the centrifuge vials and spun at 1200 r.p.m. for 5 min. The supernatant was discarded and the cells were resuspended in 0.2% trypan blue (Gibco). Viability was assessed by counting the proportion of viable cells in a haemocytometer.

### Statistics

The statistical significance of differences between control and treatment groups was determined using the two-tailed unpaired t-test. Significance was defined as $P < 0.05$.

### RESULTS

### NOS activity

Calcium-dependent NOS activity was found in the mucosa of normal urinary bladder biopsy specimens, but no calcium-independent activity was detected (Table 1). Tumour tissues showed significant calcium-independent NOS activity and the calcium-dependent NOS activity was similar to the activity in normal mucosa (Table 1). In the mucosa of BCG-treated patients, there was a fourfold increase in calcium-dependent NOS activity, as compared with normal control subjects (Table 1). Patients treated with BCG also showed a high calcium-independent NOS activity (Table 1).

### Immunohistochemistry of tissue sections

In both normal urothelium (Figure 1A) and tumour tissue (Figure 1B), a marked eNOS-like immunoreactivity was seen in the urothelial cells. eNOS-like immunoreactivity was also found in BCG-treated bladder mucosa (not shown). Furthermore, eNOS-like immunoreactivity was evident in the endothelium of blood vessels (Figure 1C). nNOS-like immunoreactivity could not be detected in normal mucosa, tumour tissue or BCG-treated mucosa, although subepithelial nNOS-like immunoreactivity was detected in some nerves in the normal bladder (Figure 1D). iNOS-like immunoreactivity could not be adequately demonstrated as both the monoclonal and polyclonal iNOS antibodies used showed staining of the urothelial cells, blood vessels, neurons and macrophages, suggesting that the iNOS antibodies were non-specific and stained all NOS isoenzymes (not shown).

### Direct measurements of gaseous NO in the urinary bladder in vivo

In BCG-treated patients, the mean NO concentration in the air aspirated from the bladder was 30 times (450 ± 240 p.p.b.) higher than in control subjects (15 ± 4 p.p.b.) (Figure 2).

**Figure 1** Micrographs of human urinary bladders after processing for indirect immunofluorescence. eNOS-like immunoreactivity (arrow) in normal urothelium (**A**), tumour tissue (**B**) and endothelium of blood vessels (**C**). **D** nNOS-like immunoreactivity (small arrow) in small nerve fibres in smooth muscle cell bundles in the normal human bladder. Bar indicates 50 µm

© Cancer Research Campaign 1998

**Figure 2** NO concentrations after 5 min of incubation with air (NO < 5 p.p.b.) in the urinary bladder of five patients treated with BCG and seven untreated control subjects. All patients had negative urine cultures

### Effect of NO donors on cell viability

A dose-dependent inhibition of [$^3$H]thymidine incorporation and a reduction in total DNA content were seen when T24 and MBT-2 bladder cancer cell lines were grown in the presence of the NO-releasing compound S-nitroso-N-acetylpenicillamine (SNAP, 10–1000 μM) (Figure 3). There was no significant difference in the sensitivity of the different cell lines. The same pattern was seen using sodium nitroprusside (SNP, 0.3–1.5 mM), although a higher concentration was needed (not shown). The inhibition of cell growth was found to be partly due to decreased cell viability as assessed by the trypan blue exclusion method. After 24 h of incubation with 100 μM SNAP, the viable cell count amounted to 70% of that of control subjects (not shown). No reduction in the DNA concentration was seen when normal urothelial cells were incubated with SNAP (10–1000 μM) under the same conditions (Figure 3A). However, inhibition of [$^3$H]thymidine incorporation was seen when normal urothelial cells were incubated with SNAP at the highest concentration used (1000 μM) (Figure 3B).

### DISCUSSION

Since the first report of the use of intravesical BCG for the treatment of superficial bladder cancer (Morales et al, 1976), clinical trials have confirmed that BCG is an effective immunomodulator that yields superior results to those of chemotherapy (Lamm, 1992). It is now apparent that BCG exerts its effect both directly on the tumour cells and via the immune system (Jackson and James, 1994). As an increase in cytokine production has been demonstrated after BCG instillations (Ratliff et al, 1986; Fleischmann et al, 1989; Jackson et al, 1995) and cytokines may cause a sustained release of high concentrations of NO, resulting in cytostatic and cytotoxic effects on tumour cells (Hibbs et al, 1990), it seemed logical to investigate the possible involvement of NO in BCG-mediated anti-tumour effects. Interestingly, BCG was one of the first compounds used to induce NOS activity in murine macrophages when the activated macrophage cytotoxic effect was studied (Hibbs et al, 1990).

We found a higher degree of both calcium-dependent and calcium-independent NOS activity in the urinary bladder following BCG treatment for superficial bladder cancer. The increased calcium-dependent activity was probably due to an increased activity of eNOS, localized to the urothelial cells. Thus, the luminal

**Figure 3** Effect of NO donor addition to cell cultures. The effect on the total DNA concentration (**A**) and [$^3$H]thymidine uptake (**B**) in bladder cancer cell lines MBT-2 (○) and T24 (□) and normal cultured human urothelial cells (△) after 48 h of incubation with SNAP (10–1000 μM). Bars represent the mean ± s.e.m. for six or more different experiments and are expressed as percentages of untreated control cells

NO measured in the bladder is probably formed in the urothelium as NO produced in deeper parts of the submucosa (in subepithelial nerves) is not likely to reach the lumen as intact NO because of its short half-life in biological tissues. The luminal NO is probably produced by eNOS activity in the urothelium, as judged from our immunohistochemical findings, although a contribution of iNOS activity cannot be ruled out. The localization of the calcium-independent NOS activity could not be assessed by immunohistochemistry, however, because of cross-reactivity of the iNOS antibodies used with all isoenzymes. It is interesting that the calcium-dependent NOS activity was induced by BCG treatment as calcium-dependent NOS is usually regarded as being constitutively expressed, in contrast to the inducible calcium-independent NOS.

There are, however, reports on cytokine induced calcium-dependent NOS activity in endothelial cells (Rosenkrantz-Weiss, 1994). It has also been shown that oestrogen increases the calcium-dependent NOS activity in various tissues, including the urinary bladder (Weiner et al, 1994; Ehrén et al, 1995). The mechanism underlying the increase in calcium-dependent and calcium-independent NOS activity after BCG treatment is not clear at the present time. Both an increase in the amount of NOS present and changes in co-factor concentrations could be possible explanations, as indicated by previous studies (Rosenkrantz-Weiss, 1994; Weiner et al, 1994). Although it is clear that NOS activity in the bladders of BCG-treated patients results in a high local concentration of NO, it is still not clear whether it is calcium-dependent or calcium-independent NOS activity that is responsible for the increased production of NO in vivo. Thus, in vitro, the calcium-dependent NOS activity dominated over the calcium-independent activity, but this does not necessarily reflect the actual in vivo situation.

Significant calcium-independent activity in bladder tumours was seen, whereas no calcium-independent activity was detected in the normal bladder mucosa. Thus, in bladder tumours, both calcium-dependent and calcium-independent activity was found. This is not surprising as it has been shown that several tumour cell lines express NOS (Radomski et al, 1991; Jenkins et al, 1995), and NOS is also present in human ovarian, uterine and breast cancer (Thomsen et al, 1994, 1995). However, the role of NO production in tumour growth is unclear. It has been shown that the NOS activity in the tumour is correlated with the tumour growth rate (Jenkins et al, 1995). Furthermore, NO may also be important for maintaining the blood supply to the tumour by dilating the malignant vessels (Andrade et al, 1992). In low concentrations, NO can stimulate cell growth, whereas high concentrations inhibit cell growth (Thomae et al, 1995). Thus, NO can be both beneficial and harmful to tumour growth. Although we do not know the actual local NO concentration in the bladder wall, our data definitely show a marked increase in the NO concentration after BCG treatment for bladder cancer. In the present study, we found pronounced inhibitory effects on cell growth and cell viability when NO donors were applied to the tumour cell lines, indicating that NO, at high local concentrations may inhibit tumour growth in bladder cancer.

We have presented a new technique for measuring NO production in the urinary bladder in vivo. This technique is very interesting as it opens up a minimally invasive possibility of measuring NO formation directly in patients with bladder cancer. We are now engaged in measurements to establish a possible relationship between NO formation and the response to BCG therapy. Furthermore, the possibility to enhance the BCG-induced NO formation by adding L-arginine will also be investigated as this may enhance the anti-tumour effect of BCG treatment.

In conclusion, we have demonstrated the presence of NOS activity in the human urinary bladder. This activity is markedly enhanced by BCG treatment and large amounts of NO are formed in the bladder during treatment. NO exerts strong inhibitory effects on the growth of bladder cancer cell lines. The present data suggest that NO may be involved in the anti-tumour activity of BCG.

### ACKNOWLEDGEMENTS

This project was supported by the Swedish MRC (project 11199), Swedish Cancer Society (project 3769-B96-OXAB), Maud and Briger Gustavsson's foundation and the Swedish foundation of Medical Research.

### REFERENCES

Andrade SP, Hart IR and Piper PJ (1992) Inhibitors of nitric oxide synthase selectively reduce flow in tumour-associated neovasculature. *Br J Pharmacol* **107**: 1092–1095

Cilento BG, Freeman MR, Schneck FX, Retik AB and Atala A (1994) Phenotypic and cytogenic characterization of human bladder urothelia expanded in vitro. *J Urol* **152**: 665–670

Ehrén I, Hammarström M, Adolfsson J and Wiklund NP (1995) Induction of calcium-dependent nitric oxide synthase by sex hormones in the guinea-pig urinary bladder. *Acta Physiologica Scand* **153**: 393–394

Farias-Eisner R, Sherman MP, Aerberhard E and Chaudhuri G (1994) Nitric oxide is an important mediator for tumoricidal activity in vivo. *Proc Natl Acad Sci USA* **91**: 9407–9411

Fleischmann JD, Toossi Z, Ellner JJ, Wentworth DB, Ratliff TL and Imbemo AL (1989) Urinary interleukins in patients receiving intravesical bacillus Calmette-Guérin therapy for superficial bladder cancer. *Cancer* **64**: 1447–1454

Hibbs JB Jr, Taintor RR, Vavrin Z, Granger DL, Drapier J-C, Amber IJ and Lancaster JR Jr (1990) Synthesis of nitric oxide from a terminal guanidino nitrogen atom of L-arginine: a molecular mechanism regulating cellular proliferation that targets intracellular iron. In *Nitric oxide from L-arginine: a bioregulatory system: Proceedings of the Symposium on Biological Importance of Nitric Oxide*, Moncada S and Higgs A (eds), pp. 189–223. Elsevier: Amsterdam

Jackson AM and James K (1994) Understanding the most successful immunotherapy for cancer. *The Immunologist* **2**: 208–215

Jackson AM, Alexandroff AB, Kelly RW, Skibinska A, Esuvaranathan K, Prescott S, Chisholm GD and James K (1995) Changes in urinary cytokines and soluble intercellular adhesion molecule-1 (ICAM-1) in bladder cancer patients after bacillus Calmette-Guérin (BCG) immunotherapy. *Clin Exp Immunol* **99**: 369–375

Jenkins DC, Charles IG, Thomsen LL, Moss DW, Holmes LS, Baylis SA, Rhodes P, Westmore K, Emson PC and Moncada S (1995) Roles of nitric oxide in tumor growth. *Proc Natl Acad Sci USA* **92**: 4392–4396

Knowles RG and Moncada S (1994) Nitric oxide synthases in mammals. *Biochem J* **298**: 249–258

Lamm DL (1992) Optimal BCG treatment of superficial bladder cancer as defined by American trials. *Eur Urol* **21**: 12–16

Moncada S and Higgs A (1995) Molecular mechanisms and therapeutic strategies related to nitric oxide. *FASEB J* **9**: 1319–1330

Morales A, Eidinger D and Bruce AW (1976) Intracavitary bacillus Calmette-Guérin in the treatment of superficial bladder tumors. *J Urol* **116**: 180–183

Radomski MW, Jenkins DC, Holmes L and Moncada S (1991) Human colorectal adenocarcinoma cells: differential nitric oxide synthesis determines their ability to aggregate platelets. *Cancer Res* **51**: 6073–6078

Ratliff TL, Haaf EO, Catalona WJ (1986) Interleukin-2 production during intravesical bacillus Calmette-Guérin therapy for bladder cancer. *Clin Immmunol Immunopathol* **40**: 375–379

Rosenkrantz-Weiss P, Sessa WC, Milstien S, Kaufman S, Watson CA and Pober JS (1994) Regulation of nitric oxide synthesis by proinflammatory cytokines in human umbilical vein endothelial cells. *J Clin Invest* **93**: 2236–2243

Thomae KR, Nakayama DK, Billiar TR, Simmons RL, Pitt BR and Davies P (1995) The effect of nitric oxide on fetal pulmonary artery smooth muscle growth. *J Surg Res* **59**: 337–343

Thomsen LL, Lawton FG, Knowles RG, Beesley JE, Riveros-Moreno V and Moncada S (1994) Nitric oxide synthase activity in human gynecological cancer. *Cancer Res* **54**: 1352–1354

Thomsen LL, Miles DW, Happerfield L, Bobrow LG, Knowles RG and Moncada S (1995) Nitric oxide synthase activity in human breast cancer. *Br J Cancer* **72**: 41–44

Weiner CP, Lizasoain I, Baylis SA, Knowles RG, Charles IG and Moncada S (1994) Induction of calcium-dependent nitric oxide synthase by sex hormones. *Proc Natl Acad Sci USA* **91**: 5212–5216

Wingo PA, Tong T and Bolden S (1995) Cancer statistics. *Cancer J Clin* **45**: 8–30

© Cancer Research Campaign 1998